# The Mediating Role of Emotional Intelligence in the Relationship Between Parental Overprotection and Offspring’s Physical Health in Adulthood

**DOI:** 10.3390/jintelligence14010001

**Published:** 2025-12-22

**Authors:** Huanhua Lu, Yawen Zhao, Zaina Jianaer, Ruihan Chen

**Affiliations:** School of Marxism, China University of Geosciences (Beijing), Beijing 100083, China

**Keywords:** parental overprotection, trait emotional intelligence, physical health, mediation analysis

## Abstract

Parental overprotection before adulthood can have enduring consequences for offspring, yet the mechanisms underlying its association with adult physical health are not fully understood. This study proposes trait emotional intelligence (trait-EI) as a pivotal mediating factor in this relationship. A sample of 459 university students (mean age = 22.42 years, SD = 1.43; 50.3% female, 49.7% male) completed measures assessing their recalled parental overprotection, trait-EI and physical health. Results from regression and mediation analyses revealed that parental overprotection was significantly negatively associated with both overall trait-EI and physical health. Critically, trait-EI was found to be a significant mediator, indicating that overprotective parenting impedes the development of trait-EI, which in turn translates into poorer health outcomes. Further analysis of the facets of trait-EI demonstrated that the intrapersonal and stress management dimensions were unique contributors to physical health, whereas interpersonal and adaptability skills were not. What’s more, a moderated mediation analysis showed that gender significantly moderated the pathway from parental overprotection to trait-EI, with the negative effect of overprotection on trait-EI being substantially stronger for male than for female offspring. These findings underscore the role of trait-EI as a central psychological mechanism translating early parenting experiences into long-term physical health and point to the need for gender-sensitive approaches in preventive health interventions.

## 1. Introduction

In modern society, parental protection of children has reached an unprecedented level. More and more parents see themselves as “guardians” in their children’s lives, aiming to create a worry-free and secure environment for their growth. However, this protective approach sometimes escalates into excessive intervention, forming what is known as “parental overprotection”. Parental overprotection refers to excessive parental intervention and control that restricts children’s independence and autonomy, while striving to eliminate all challenges and difficulties they may face (Barber, 1996; Thomasgard & Metz, 1993; Thomasgard et al., 1995). Through excessive intervention and control, parents attempt to shield their children from unpleasant emotional experiences or life challenges. However, although parental overprotection often stems from genuine care and protective intentions, it is increasingly recognized as a parenting approach that may restrict children’s autonomy and adaptive development, leading to various unintended challenges for their emotional and psychological growth.

A substantial number of studies have demonstrated that parental overprotection is closely associated with emotional distress and psychological difficulties in children and adolescents, including anxiety, depression, poor emotion regulation, and heightened stress sensitivity (Carollo et al., 2024; de Roo et al., 2022; Kim, 2019; Lee et al., 2022; Li et al., 2025; Manuele et al., 2023; Van Petegem et al., 2020; Van Petegem et al., 2022; Yu et al., 2024). For example, Li et al. (2025) found that overprotective parenting negatively affects children’s emotional regulation abilities, which in turn leads to higher levels of anxiety and depressive symptoms. Similarly, Barber (1996) pointed out that parental overprotection hindered the development of children’s emotional independence by depriving them of opportunities for autonomous decision-making, weakened their emotion regulation abilities, and ultimately led to maladaptive anxiety responses when individuals face challenges.

However, although the impact of parental overprotection on mental and emotional outcomes is well established (e.g., Carollo et al., 2024; de Roo et al., 2022; Kim, 2019), much less is known about its implications for long-term physical health—representing an important yet underexplored research gap. Theoretical frameworks and emerging empirical evidence suggest multiple pathways through which parental overprotection may compromise offspring’s physical health. First, according to the stress–health model, chronic exposure to stress within an overcontrolling family environment may activate physiological dysregulation, including immune suppression, endocrine disturbance, and systemic inflammation, thereby increasing risk for physical illness (Shields & Slavich, 2017; Slavich, 2016). Second, based on social support theory, parental overprotection may restrict children’s opportunities to independently build social networks and develop interpersonal coping strategies, increasing loneliness and social isolation—both established risk factors for poor physical health (Ahlich et al., 2020; Birmingham & Holt-Lunstad, 2018). Third, following health behavior theory, overprotective parenting may limit opportunities for children to learn self-management and health maintenance skills, fostering unhealthy lifestyle patterns such as sedentariness and inadequate diet, which contribute to long-term health risk (Glanz et al., 2008; Rubinelli & Diviani, 2020). Supporting these theoretical pathways, recent findings have shown that parental overprotection is associated with impaired sleep quality (Pizzo et al., 2022) and dysregulation of the hypothalamic–pituitary–adrenal (HPA) axis (Chen et al., 2022), indicating potential biological mediators of adverse health outcomes. Yet, despite these plausible mechanisms and emerging evidence, surprisingly few empirical studies have directly examined the association between parental overprotection and physical health in adulthood or identified the psychological mechanisms underlying this association. Addressing this gap constitutes the novel contribution of the present study.

A promising psychological mechanism that may translate early experiences of overprotection into long-term physical health outcomes is emotional intelligence (EI). EI refers to an individual’s ability to recognize, understand, and manage their own and others’ emotions, thereby enabling more effective environmental adaptation, problem-solving, and the enhancement of personal and social functioning (Bar-On, 1997; Perloff, 1997; Salovey & Mayer, 1990). The Bar-On model of EI posits that EI encompasses a multidimensional set of competencies, including intrapersonal skills (e.g., emotional self-awareness), interpersonal skills, adaptability, and stress management (Bar-On, 1997, 2006). It further highlights that EI is not a fixed trait—its development can be influenced by education, training, and personal effort (Bar-On, 1997, 2006). This model is particularly suitable for the present investigation as it provides a comprehensive framework for assessing the broad range of emotional and social competencies that are likely shaped by parenting styles and are relevant for health maintenance. Parental overprotection, characterized by decision substitution, extreme risk aversion, and deprivation of autonomous exploration (Barber, 1996; Thomasgard & Metz, 1993; Thomasgard et al., 1995), may critically impede the development of these EI competencies. By excessively intervening in a child’s behaviors and emotional responses, overprotective parenting can hinder the development of emotional self-awareness, limit opportunities to acquire emotion regulation strategies, restrict practice in independent decision-making and problem-solving (undermining adaptability), and reduce chances for autonomous social interaction, potentially stunting empathy and interpersonal skills (Barber & Harmon, 2002; Bar-On, 1997, 2006). Consistent with this, previous studies have found that parental overprotection negatively predicts EI in children and adolescents (Arslan et al., 2023; Gao et al., 2024; Gugliandolo et al., 2019; Wu et al., 2021), though its association with EI in adulthood is less clear.

On the other hand, EI is recognized as a critical factor in promoting physical health (Keefer et al., 2009; Sarrionandia & Mikolajczak, 2020; van Heck & den Oudsten, 2008; Woolery & Salovey, 2004; Zeidner et al., 2006). Individuals with high EI can more accurately identify stress signals and employ adaptive strategies (e.g., problem-solving, cognitive reappraisal, seeking support) to manage stress, thereby reducing the intensity and frequency of physiological stress responses and safeguarding bodily systems from excessive strain (van Heck & den Oudsten, 2008). High-EI individuals, through enhanced self-management, directly foster beneficial health behaviors (e.g., adequate sleep, balanced nutrition, regular exercise, smoking/alcohol avoidance) while minimizing risky ones, thus supporting physical health (Zeidner et al., 2006). Furthermore, those with high EI possess strong empathy and interpersonal skills, enabling them to build robust social networks that contribute to health maintenance (van Heck & den Oudsten, 2008; Zeidner et al., 2006). Effective emotion regulation, another hallmark of high EI, helps sustain physiological homeostasis and reduces psychosomatic symptoms (e.g., chronic pain, fatigue, gastrointestinal issues) (Woolery & Salovey, 2004). Prior cross-sectional studies have confirmed a significant positive correlation between general EI and self-rated physical health (Baudry et al., 2018; Espinosa & Kadić-Maglajlić, 2018; Licata et al., 2023; Mérida-López et al., 2019; Moradian et al., 2022; Peláez-Fernández et al., 2022; K. Wang et al., 2022), though the association between EI subdimensions and physical health is less clear. Therefore, EI may serve as an important mediator linking parental overprotection with offspring’s physical health.

Gender and socioeconomic status (SES) are well-established determinants of physical health and emotional development and therefore deserve attention in studies examining health-related mechanisms. SES is one of the strongest predictors of physical health, influencing health outcomes through pathways such as access to resources, health behaviors, lifestyle limitations, and cumulative exposure to chronic stress (Adler et al., 2016). Additionally, gender differences in parental socialization practices may shape how children respond to parenting behaviors such as overprotection, potentially leading to different developmental outcomes (Endendijk et al., 2016). Considering these findings, the present study controlled for SES and gender in all analyses to isolate the unique associations among parental overprotection, EI and physical health.

In summary, while extant research has established a significant link between parental overprotection and offspring’s mental health, considerable gaps remain in understanding its long-term implications for physical health and the underlying psychological mechanisms. This study, therefore, aims to address these gaps by testing whether parental overprotection before adulthood was associated with adult physical health and whether EI mediates this relationship. We hypothesized that: (1) parental overprotection was negatively associated with adult physical health; (2) EI mediates the association between parental overprotection and physical health. The novelty and significance of our research are twofold: (1) It shifts the focus from the impact of parental overprotection on mental health to the examination of its long-term physical health consequences, which will provide a more integrated perspective on how the early family environment shapes lifelong health; (2) It systematically incorporates EI and its subdimensions into the relationship between parental overprotection and physical health in young adulthood. This approach not only clarifies how the effect occurs but also identifies which specific emotional competencies are most pivotal, thereby offering precise targets for intervention.

## 2. Method

### 2.1. Participants

A priori power analysis was conducted using G*Power 3.1 to determine the required sample size. Based on α = 0.05, effect size f^2^ = 0.05 (indicating a small effect size; Cohen, 1988), and five predictors in the regression model (parental overprotection, emotional intelligence, and three control variables), a minimum sample size of 402 participants was required to achieve 95% statistical power. To ensure sufficient statistical robustness and account for potential attrition across multi-wave data collection, we initially recruited 500 participants.

Participants were recruited through snowball sampling, which was chosen due to the difficulty of obtaining geographically diverse university student samples through random sampling and because this method facilitates participant retention in multi-wave survey designs. Inclusion criteria were: (a) age ≥ 18 years, (b) currently enrolled as a university student residing in China, and (c) self-reported absence of cognitive or neurological conditions (e.g., traumatic brain injury, clinically diagnosed psychiatric disorders). Exclusion criteria included: (a) missing more than 8% of responses, (b) uniform responding across all items (e.g., selecting the same option for every item), (c) patterned responding (e.g., repetitive sequences such as “1-2-3-4-1-2-3-4”), and (d) abnormally short completion time, substantially shorter than the minimum expected response duration.

A total of 500 university students initially participated in the study. Each participant completed three survey sessions separated by two-month intervals. After excluding invalid responses based on the above criteria and removing participants who withdrew during the survey process, 459 valid cases were retained for analysis. The basic information of the participants is presented in Table 1. Marital status is not reported as the sample was predominantly unmarried/unpartnered university students. This study received ethical approval from the China University of Geosciences (Beijing) Psychological Ethics Committee (NO.20240105). All participants voluntarily took part in the study and signed an informed consent form prior to the survey.

### 2.2. Measures

Parental overprotection. Parental overprotection was assessed as retrospective perceptions of participants’ experiences of parental behavior before adulthood. Participants completed the Parental Overprotection subscale of the Parental Bonding Instrument (PBI; Parker et al., 1979), reporting how they remembered their parents’ behavior during their childhood and adolescence. Thus, the variable represents perceived parental overprotection rather than an objective historical record. Although retrospective perception measures may contain recall bias, they capture individuals’ subjective experience of parenting, which is considered particularly relevant because perceived parental behaviors more strongly predict developmental outcomes than objective records (Murphy et al., 2010). Although the PBI was originally developed in 1979, it remains widely used at present and demonstrates stable psychometric performance across cultures (e.g., Arslan et al., 2023; Chen et al., 2022; Gao et al., 2024). The PBI was translated and culturally adapted into Chinese following the standard translation and back-translation procedure and has demonstrated good psychometric properties among Chinese adult populations (e.g., Chen et al., 2022). Participants were instructed to recall typical parenting experiences that occurred before the age of 18. The instruction was as follows: “*Please recall your typical experiences with your parents before age 18, and rate each item according to how your parents behaved most of the time during that period*.” The overprotection subscale consists of 13 items rated on a 4-point Likert scale (0 = very unlike, 3 = very like). A sample item is: “My father/mother tried to control everything I did”. In the present study, Cronbach’s α for the overprotection scale was 0.88. Maternal and paternal overprotection scores were combined to generate a total parental overprotection score for inferential analyses.

Physical health. Physical health was measured using the physical-symptom items of Chinese Constitution Questionnaire (CCQ) (Q. Wang et al., 2006). Previous studies have shown that the scale has good reliability and validity and can be used as an indicator to assess an individual’s health status (Lu et al., 2018; Song et al., 2015; Q. Wang et al., 2006; Zhu et al., 2007, 2008). The CCQ consists of 60 items, including 51 items measuring physical health and 9 items measuring mental health. Because the focus of the present study was physical rather than psychological health outcomes, items measuring mental health were excluded. That is, the total score of the 51 physical-symptom items was calculated as an indicator of physical health. The questionnaire uses a 5-point Likert scale (1 = never, 5 = always). A sample item is: “Are you more susceptible to catching colds than other people?”. In this sample, Cronbach’s α was 0.95, supporting adequate internal consistency.

EI. Trait EI was assessed using the Chinese version of the Bar-On Emotional Quotient Inventory (EQ-i; Bar-On, 1997). The Chinese EQ-i has demonstrated good reliability and validity in Chinese cultural contexts (Song et al., 2015). The EQ-i includes six dimensions: Intrapersonal competence, Interpersonal competence, Adaptability, Stress Management, General Mood, and Validity. In the present study, only the first four core ability-related dimensions were used to represent emotional intelligence. The General Mood subscale was excluded because it primarily reflects emotional state rather than emotional ability (Bar-On, 1997), and many previous studies have similarly removed this dimension when conceptualizing EI as a competence construct rather than an affective disposition (e.g., Mikolajczak et al., 2009; Petrides et al., 2016; Petrides & Furnham, 2001). The Validity dimension serves to detect response consistency and does not represent emotional competencies; therefore, it was also excluded. The total score of the remaining four dimensions (Intrapersonal competence, Interpersonal competence, Adaptability, Stress Management) was computed as the indicator of EI. The EQ-i consists of 133 items, rated on a 5-point Likert scale (1 = very inconsistent, 5 = very consistent). An example item is: “I am good at understanding how other people feel”. In this sample, the Cronbach’s α coefficients were: Intrapersonal = 0.92, Interpersonal = 0.87, Stress Management = 0.89, and Adaptability = 0.84, indicating good internal consistency. The Bar-On EQ-i was selected over ability-based (e.g., MSCEIT) or personality-oriented EI measures (e.g., TEIQue) because it focuses specifically on emotional and social competencies that enable effective stress regulation, emotional management, and adaptability, which are strongly linked to physiological functioning and physical health outcomes (O’Connor et al., 2019). Prior studies have demonstrated that EQ-i dimensions predict stress-related biomarkers, health behaviors, and somatic symptoms (e.g., Mikolajczak et al., 2007; Mikolajczak et al., 2009), making it theoretically well aligned with the aims of the present study, which examines emotional competence as a mechanism through which early parenting experiences are associated with adult physical health.

Participants’ basic information. Participants’ basic information including gender, age, and family SES was obtained. Family SES was assessed using the MacArthur Scale of Subjective Social Status (family version) (Adler et al., 2000). This measure consists of a single visual ladder with 10 rungs, where higher rungs represent higher levels of socioeconomic standing. Participants were instructed to imagine the ladder as representing the distribution of families in society and to select the rung that best reflected their own family’s position in terms of income, education, and occupational prestige. Responses ranged from 1 (lowest standing) to 10 (highest standing), with higher scores indicating higher perceived family SES. The MacArthur Scale of Subjective Social Status has been widely validated and is commonly used as a reliable single-item indicator of subjective SES in psychological and health research (Adler et al., 2000; Operario et al., 2004; Singh-Manoux et al., 2005).

### 2.3. Procedure

Each participant completed three survey sessions: in the first session, participants recalled their pre-adulthood experiences with parental upbringing and completed the Parental Overprotection subscale of the PBI (Parker et al., 1979); after a two-month interval, they completed the EQ-i (Bar-On, 1997) during the second session; following another two-month interval, participants completed the CCQ (Q. Wang et al., 2006) in the third session.

To protect anonymity and ensure secure tracking across the three waves, participants were instructed to generate a unique anonymous nickname known only to themselves and to use it consistently in each survey session. This anonymous code allowed data matching across the three waves without exposing any personal identity. In addition, participants were asked to provide an email address for the purpose of receiving follow-up contact and survey reminders. The email account did not contain personally identifiable information (e.g., names, student ID numbers), and participants were encouraged to use non-identifiable email formats (e.g., randomly generated character addresses). The email addresses were stored separately from survey responses, used exclusively for longitudinal follow-up communication, and were not linked to questionnaire data in the dataset. No other personal identifiers (such as names or phone numbers) were collected at any stage. All data were stored on encrypted, password-protected institutional servers with access limited to authorized research team members.

This three-wave data collection procedure was adopted for two primary methodological reasons. First, the temporal separation of the independent variable (parental overprotection), mediator (EI), and dependent variable (physical health) allowed us to establish a logical time-ordered framework that strengthens the theoretical plausibility of the hypothesized mediation model. Although the design cannot confirm causal inference, it offers a more robust analytical strategy compared with traditional single-wave cross-sectional studies, where temporal precedence is assumed rather than empirically structured. Second, the multi-wave design serves as an effective procedural remedy to reduce common method bias (Podsakoff et al., 2003). When all constructs are assessed concurrently from the same respondents, the associations among variables may be artificially inflated by response consistency tendencies, item context effects, or temporary affective states. Separating data collection points will help mitigate these threats to validity.

The two-month interval was chosen as an empirically reasonable and practically feasible duration. It is sufficiently long to minimize carryover effects and reduce participants’ memory of previous survey responses (Dillman et al., 2014), while also short enough to maintain participant retention throughout the study. Similar multi-wave psychological and behavioral studies have adopted comparable 1–3-month lags to examine temporal processes and test mediation models (e.g., Allan & Blustein, 2022; Rijnhart et al., 2022; Selig & Preacher, 2009).

Nevertheless, we acknowledge that the present design does not include repeated measurement of all variables across waves, preventing cross-lagged panel analysis and thus limiting our ability to draw causal conclusions.

### 2.4. Statistical Analysis

Data analysis followed a structured sequence to test our research hypotheses. First, descriptive statistics and correlation analysis were computed to examine the basic relationships among variables. Second, three hierarchical regression analyses were conducted for three specific purposes: (1) to examine the relationship between parental overprotection and physical health after accounting for demographics (gender, age and family SES); (2) to examine the relationship between parental overprotection and overall EI after accounting for demographics; and (3) to examine the relationship between the four EI subdimensions and physical health after accounting for both demographics and the other subdimensions. Third, to formally test our main hypothesis that EI mediates the parental overprotection-physical health relationship, we conducted mediation analysis using Model 4 of the PROCESS macro for SPSS 27 with 5000 bootstrap samples. This analysis provided a direct test of the indirect effect while controlling for the same demographic covariates. Finally, to examine whether the mediation model differed across gender and family economic status, we conducted moderated mediation analyses using the PROCESS macro (Model 59; Hayes, 2018). Model 59 allows simultaneous testing of moderation on all three components of the mediation model: the association between parental overprotection and overall EI (a-path), the association between overall EI and physical health (b-path), and the direct association between parental overprotection and physical health (c′-path). Gender and family economic status were entered separately as moderators. For significant interaction effects, simple slope analyses were conducted to interpret moderation patterns. Conditional indirect effects were estimated using 5000 bootstrap samples, and 95% bootstrap confidence intervals were calculated to determine statistical significance. All analyses were preceded by checks for statistical assumptions, including multicollinearity (all VIFs < 5) and normality of residuals.

## 3. Results

### 3.1. Descriptive Analysis

Table 2 displays the descriptive statistics and gender differences for the major variables. For each measure, means and standard deviations were reported separately for males and females, along with *t*-test results and effect sizes (Cohen’s d). All variables demonstrated acceptable levels of normality, with absolute skewness and kurtosis values below 1, indicating approximate normal distributions suitable for subsequent regression and mediation analyses. The independent samples *t*-test revealed a significant gender difference in interpersonal competence (*t* = −3.305, *p* = 0.001) which was a sub-dimension of EI, while no significant gender differences were found for the remaining variables.

### 3.2. Common Method Bias Test

To examine potential common method bias, Harman’s single-factor test was performed (Podsakoff et al., 2003). The first factor explained only 37.24% of the total variance, which is below the recommended threshold of 40%, suggesting that common method bias was not a significant concern in this study.

### 3.3. The Relationship Between Parental Overprotection, Offspring’s Physical Health, and EI

Correlation analysis (Table 3) showed that the key study variables were significantly associated in the expected directions. Higher levels of parental overprotection were related to lower trait EI and poorer physical health, whereas greater trait EI—both overall and across its four sub-dimensions—was associated with better physical health. These patterns are consistent with our hypotheses and provide empirical justification for conducting the subsequent regression and mediation analyses.

This study employed hierarchical regression analysis to examine the relationship between parental overprotection and offspring’s physical health. Taking offspring’s physical health as the dependent variable, the first layer included demographic variables (offspring’s age, offspring’s gender, and family SES) as predictor variables; the second layer incorporated parental overprotection as predictor variables. The results showed that after adding parental overprotection, the explanatory power of the regression model significantly increased (*β* = −0.205, *t* = −4.449, *p* < 0.001, Δ*R*^2^ = 0.041, Δ*F* (1, 456) = 19.792, *p* < 0.001, Table 4), indicating that parental overprotection is a risk factor for offspring’s physical health.

Similarly, another hierarchical regression analysis was conducted to examine the relationship between parental overprotection and offspring’s overall EI. The results showed that parental overprotection was significantly associated with offspring’s overall EI (*β* = −0.302, *t* = −6.744, *p* < 0.001, Table 5), indicating that parental overprotection is a risk factor for offspring’s overall EI.

A hierarchical regression analysis was conducted to examine the relationship between the four sub-dimensions of EI and physical health. Taking physical health as the dependent variable, the first layer included demographic variables (age, gender, and family SES) as predictor variables; the second layer incorporated the four sub-dimensions of EI as predictor variables. The results showed that after adding the four sub-dimensions of EI, the explanatory power of the regression model significantly increased (Δ*R*^2^ = 0.238, Δ*F* (4, 456) = 36.072, *p* < 0.001, Table 6), indicating that the four sub-dimensions of EI as a whole had a significant association with physical health. The results also found that after controlling for demographic variables and other three sub-dimensions of EI, stress management was significantly associated with physical health (*β* = 0.175, *t* = 2.562, *p* = 0.011, Table 6), and intrapersonal competence was also significantly associated with physical health (*β* = 0.220, *t* = 2.848, *p* = 0.005, Table 6); however, after controlling for demographic variables and other three sub-dimensions of EI, adaptability was not significantly associated with physical health (*β* = 0.111, *t* = 1.316, *p* = 0.189, Table 6), and interpersonal competence also was not significantly associated with physical health (*β* = 0.038, *t* = 0.647, *p* = 0.518, Table 6). This reveals that stress management and intrapersonal competence can independently and positively be associated with physical health.

### 3.4. Mediation Analysis

**Figure 1:**
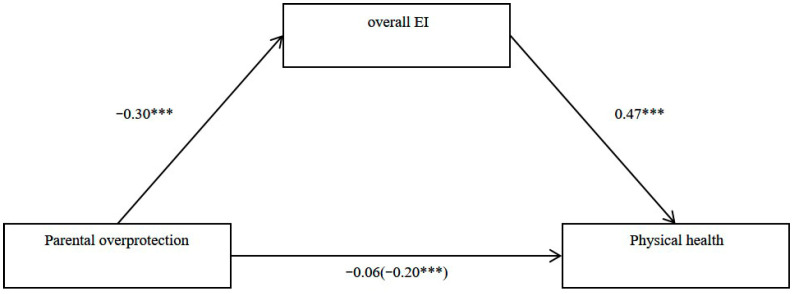
EI mediated the relationship between parental overprotection and physical health. The values beside each arrow were the path coefficients of the mediated analysis, after controlling for participants’ gender, age, and family SES. For the association between parental overprotection and physical health, the value in brackets is the total correlation, and the value outside of brackets is the correlation after being mediated by overall EI. All values are standardized betas. *** *p* < .001. EI = Emotional intelligence.

To examine the mediating role of offspring’s overall EI between parental overprotection and offspring’s physical health, a mediation analysis was conducted using PROCESS model 4, with parental overprotection as the independent variable, offspring’s Overall EI as the mediator, offspring’s physical health as the dependent variable, and offspring’s age, offspring’s gender, and family SES as control variables. The results showed that the total effect of parental overprotection on offspring’s physical health was significant (*β* = −0.205, *t* = −4.449 *p* < 0.001, 95% CI [−0.753, −0.292], Figure 1). After including the offspring’s overall EI as a mediator, the direct effect of parental overprotection on offspring’s physical health became non-significant (*β* = −0.064, *t* = −1.475, *p* = 0.14, 95% *CI* [−0.378, 0.054], Figure 1). The bootstrap simulation (n = 5000) indicated that the indirect effect through the offspring’s total EI was significant (*b* = −0.361, Boot SE = 0.065, 95% Boot CI [−0.494, −0.241]). This indirect effect accounted for approximately 68.78% of the total effect. These results indicated that offspring’s overall EI mediated the relationship between parental overprotection and offspring’s physical health.

### 3.5. Parallel Mediation Analyses

**Figure 2:**
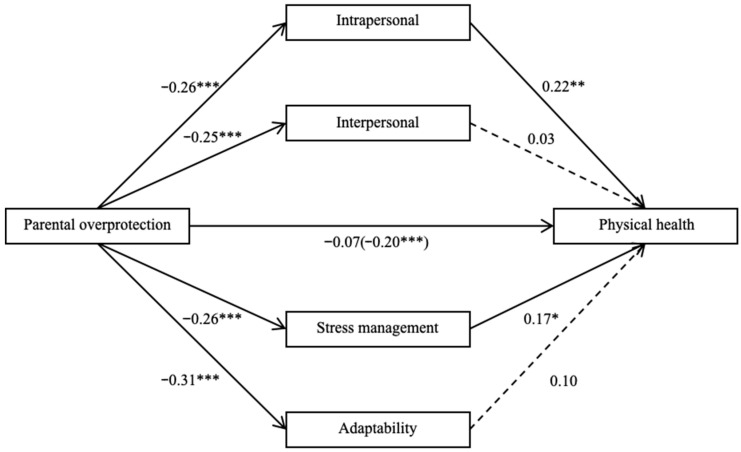
Sub-dimensions of EI mediated the relationship between parental overprotection and physical health. The values beside each arrow were the path coefficients of the mediated analysis, after controlling for participants’ gender, age, and family SES. For the association between parental overprotection and physical health, the value in brackets is the total correlation, and the value outside of brackets is the correlation after being mediated by sub-dimensions of EI. All values are standardized betas. * *p* < .05, ** *p* < .01, *** *p* < 0.001. EI = Emotional intelligence.

To examine the mediating role of the four sub-dimensions of EI, a parallel mediation analyses were conducted, using parental overprotection as independent variables, the four sub-dimensions of offspring’s EI (intrapersonal competence, interpersonal competence, stress management, and adaptability) as parallel mediators, offspring’s physical health as the dependent variable, and offspring’s age, offspring’s gender, and family SES as control variables. The results showed that the total effect of parental overprotection on offspring’s physical health was significant (*β* = −0.205, *t* = −4.449, *p* < 0.001, 95% CI [−0.753, −0.292], Figure 2). After including the four sub-dimensions of offspring’s EI as mediators, the direct effect of parental overprotection on offspring’s physical health became non-significant (*β* = −0.066, *t* = −1.533, *p* = 0.126, 95% CI [−0.386, 0.048], Figure 2). The bootstrap simulation (n = 5000) demonstrated that the total indirect effect through all four mediators was significant (*b* == −0.353, Boot SE = 0.068, 95% Boot CI [−0.495, −0.231]). This total indirect effect accounted for approximately 67.80% of the total effect. Specifically, intrapersonal competence (*b* == −0.145, Boot SE = 0.058, 95% Boot CI [−0.264, −0.040]) and emotion regulation (*b* == −0.113, Boot SE = 0.053, 95% Boot CI [−0.229, −0.018]) exerted significant mediating effects. In contrast, the mediating effects of interpersonal competence (*b* == −0.020, Boot SE = 0.043, 95% Boot CI [−0.105, 0.064]) and adaptability (*b* == −0.075, Boot SE = 0.068, 95% Boot CI [−0.215, 0.057]) were not significant.

### 3.6. Moderated Mediation Analysis

**Figure 3:**
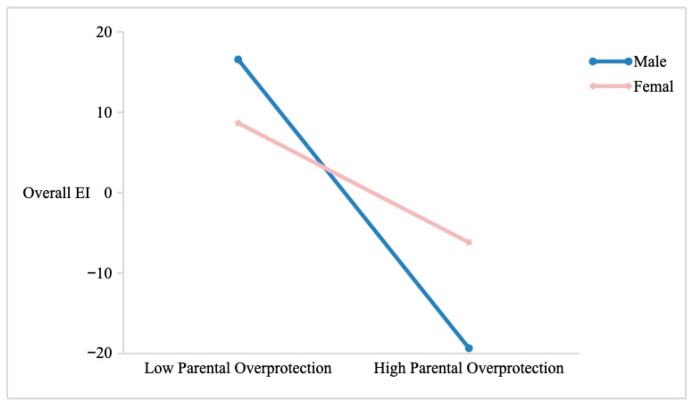
Moderating Effect of Gender on the Relationship Between Parental Overprotection and Offspring’s Overall EI.

We conducted a moderated mediation analysis using PROCESS Model 59 to test whether offspring’s gender moderated the indirect effect of parental overprotection on physical health through overall EI, with parental overprotection as the predictive variable, physical health as the predicted variable, and overall EI as the intermediary variable, gender as the moderator. The results (Table 7) showed that the interaction between parental overprotection and gender was significantly associated with overall EI (*b* = 0.912, SE = 0.330, *t* = 2.762, *p* = 0.006, 95% CI [0.263, 1.560]), but it was not significantly associated with physical health (*b* = 0.015, SE = 0.219, *t* = 0.070, *p* = 0.944, 95% CI [−0.415, 0.446]). At the same time, the interaction between overall EI and gender was also not significantly associated with physical health (*b* = 0.013, SE = 0.059, *t* = 0.215, *p* = 0.830, 95% CI [−0.103, 0.129]). A simple slope test (Figure 3) showed that for male offspring, parental overprotection was significantly associated with overall EI (*b* = −1.555, SE = 0.233, *t* = −6.660, *p* < 0.001, 95% CI [−2.013, −1.096]); while for female offspring, the association between parental overprotection and overall EI was also significant but the effect is weakened (*b* = −0.643, SE = 0.233, *t* = −2.754, *p* = 0.006, 95% CI [−1.101, −0.184]). The bootstrap analysis (n = 5000) demonstrated the mediating effect of “parental overprotection → overall EI→ physical health” was significant for both female (*b* = −0.215, Boot SE = 0.077, 95% Boot CI [−0.384, −0.077]) and male offspring (*b* = −0.500, Boot SE = 0.099, 95% Boot CI [−0.706, −0.319]). These results indicated that gender moderated the first stage of the mediation model, leading to stronger negative effects of parental overprotection on overall EI among male offspring.

Besides, we performed another moderated mediation analysis using PROCESS Model 59 to test whether family SES moderated the indirect effect of parental overprotection on physical health through overall EI. The results showed that no significant interaction was found, revealing that family SES could not moderate the mediating model of “parental overprotection → overall EI → physical health”.

## 4. Discussion

The present study examined the relationship between retrospective perceived parental overprotection before adulthood and physical health in adulthood, as well as the potential mediating role of trait EI. The findings demonstrated that parental overprotection was negatively associated with physical health, and trait EI mediated this association. Specifically, the dimensions of stress management and intrapersonal competence served as significant mediators, whereas interpersonal competence and adaptability did not show significant indirect effects. Beyond these primary mediation findings, the present study also found that gender significantly moderated the association between parental overprotection and trait EI, such that the negative effect of overprotection on trait EI was considerably stronger among male than female offspring. Taken together, these findings advance the existing literature by empirically demonstrating not only a mediating psychological mechanism linking early overprotective parenting to adult physical health but also a gender-related difference in how this developmental process unfolds.

First, this study found that individuals who recall higher levels of parental overprotection before adulthood reported poorer self-rated physical health in adulthood. One possible explanation is that parental overprotection reduces opportunities for independent coping and adaptive stress regulation, resulting in biological dysregulation and maladaptive health behaviors over time. While previous research has focused primarily on the effects of parental overprotection on mental health (Carollo et al., 2024; de Roo et al., 2022; Kim, 2019; Lee et al., 2022; Li et al., 2025; Manuele et al., 2023; Van Petegem et al., 2020; Van Petegem et al., 2022; Yu et al., 2024), the present findings extend existing knowledge by suggesting that the negative consequences of overprotective parenting may persist into adulthood and influence physical health outcomes.

More importantly, this study also found that EI mediated the negative association between parental overprotection before adulthood and offspring’s physical health in adulthood, supporting the notion that EI is a key developmental resource. To our knowledge, this is one of the first studies to explicitly identify EI as a mechanism linking parental overprotection to physical health outcomes. The exploratory analysis of EI subdimensions offers a more nuanced understanding. The fact that intrapersonal competence and stress management emerged as unique mediators, while interpersonal skills and adaptability did not, is highly revealing. This pattern suggests that the health detriment associated with overprotection stems primarily from internal deficits—namely, an impaired ability to understand and regulate one’s own emotions and to cope effectively with stress. Overprotective parenting, by solving problems for the child and shielding them from stressors, may precisely rob them of the opportunities needed to practice and develop these core intrapersonal and self-regulation skills (Barber & Harmon, 2002; Bar-On, 1997, 2006). In contrast, while overprotection might also limit social opportunities, the competencies for building interpersonal relationships might be developed and compensated for in other social contexts (e.g., school, peers), making them less central to the health pathway examined here. This delineation is a significant contribution, moving the field from a broad understanding of EI to identifying the specific emotional skills that are most vulnerable to overprotective parenting and most critical for long-term health.

Beyond the primary mediation findings, the present study found that gender significantly moderated the association between parental overprotection and trait EI, such that the negative effect of parental overprotection on trait EI was substantially stronger for male than for female offspring. This pattern suggests that men may be more vulnerable to the emotional developmental constraints imposed by overcontrolling parenting. One plausible explanation is that, in many cultural contexts, boys receive fewer opportunities and less encouragement for emotional expression and interpersonal communication than girls, making them more susceptible to parental behaviors that further limit their autonomy and emotional exploration. Consequently, parental overprotection may more strongly inhibit boys’ development of EI, which in turn increases their long-term health risks. This finding highlights the necessity of considering gender when examining the long-term effects of early parenting and underscores the need for gender-sensitive interventions aimed at strengthening EI, particularly among young men who experienced overprotective family environments.

### 4.1. Theoretical and Practical Implications

Theoretically, this study enriches the parenting literature by positioning EI as a key bridge between parental behavior and offspring’s physical health, a link less established than that with mental health. It supports and extends the stress-health model (Slavich, 2016; Shields & Slavich, 2017) by specifying a key psychological factor (EI) that translates chronic social stress (from parental overprotection) into physiological dysregulation. Furthermore, our findings regarding the specific EI subdimensions provide a refined, mechanism-driven target for preventive interventions.

Practically, these results underscore the importance of educating parents about the long-term health consequences of overprotection. Parenting programs should emphasize the value of granting age-appropriate autonomy, allowing children to experience manageable challenges, and guiding them in developing emotional awareness and coping strategies, rather than eliminating all obstacles for them. For young adults, interventions aimed at enhancing intrapersonal competence and stress management techniques (e.g., mindfulness, cognitive reappraisal training) could potentially mitigate the negative health impacts stemming from earlier overprotective experiences.

### 4.2. Limitations and Future Directions

Despite its contributions, this study has several limitations. First, although the use of multi-wave data collection reduced some limitations of cross-sectional designs, the design did not allow for cross-lagged analysis because each variable was assessed at only one time point, which prevents definitive causal inference. Future longitudinal and experimental studies are needed to rigorously examine causal directionality. Second, reliance on self-report measures may introduce potential social desirability and common method bias. Future researchers are encouraged to incorporate multi-informant reports (e.g., parents, peers) or objective biomarkers (e.g., cortisol levels, inflammatory markers) to enhance validity. Third, although gender and SES were statistically controlled, other potentially influential contextual factors such as being an only child, attachment style and personality traits were not measured and should be examined in future research. Fourth, the sample consisted of Chinese university students, limiting generalizability across ages and cultures. The impact of overprotection might vary across cultures, warranting future cross-cultural research. Fifth, the reliance on retrospective self-reports of parenting practices is subject to recall bias. Future studies would benefit from using prospective, longitudinal designs that assess parenting in real-time. Finally, we focused exclusively on EI as a mediator. Future research would benefit from incorporating additional mediators (e.g., self-efficacy, resilience, health behaviors) into comprehensive multiple-mediator or longitudinal cross-lagged models to further clarify developmental pathways.

## 5. Conclusions

In conclusion, this study demonstrates that the negative association between parental overprotection before adulthood and offspring’s physical health in adulthood is mediated by trait EI. Crucially, this effect is driven specifically by deficits in intrapersonal competence and stress management skills. Moreover, the present findings highlight an important gender-specific pattern: the detrimental impact of parental overprotection on trait EI was substantially stronger for male than for female offspring, resulting in a more pronounced indirect effect on physical health among men. Together, these findings not only advance our theoretical understanding of the long-term reach of parenting but also provide clear, actionable targets for fostering healthier emotional development and physical well-being, particularly among individuals—such as young men—who may be more vulnerable to the emotional constraints imposed by early overprotective parenting.

## Figures and Tables

**Table 1:** Participant Demographics (N = 459).

Characteristic		Value
Age	M	22.42
	SD	1.43
Gender	Male	231 (50.30%)
	Female	228 (49.70%)
Family SES	Low	50 (10.89%)
	Middle	342 (74.51%)
	High	67 (14.60%)

Note. Family socioeconomic status (SES) was measured on a 10-point scale. Participants were categorized into three groups based on their scores: Low SES (scores 1–3), Middle SES (scores 4–7), and High SES (scores 8–10).

**Table 2:** Descriptive statistics of all variables (N = 459).

Measures	Variables	Range	*Skewness*	*Kurtosis*	Gender	*M ± SD*	*t*	*p*	*Cohen’s d*
PBI	Parental overprotection	15–76	−0.115	−0.328	Male	45.34 ± 11.54	−0.471	0.638	−0.044
					Female	45.85 ± 11.61			
EQ-i	Intrapersonal competence	87–194	0.676	0.178	Male	136.29 ± 18.55	−0.057	0.955	−0.005
					Female	136.39 ± 17.62			
	Interpersonal competence	51–113	0.327	−0.059	Male	80.29 ± 9.98	−3.305	0.001	−0.309
					Female	83.48 ± 10.68			
	Stress management	20–89	0.191	0.843	Male	62.65 ± 9.33	1.746	0.082	0.163
					Female	61.18 ± 8.74			
	Adaptability	39–120	0.654	0.955	Male	80.52 ± 11.94	−0.258	0.797	−0.024
					Female	80.78 ± 9.64			
	Overall EI	205–495	0.663	0.401	Male	359.74 ± 45.02	−0.516	0.606	−0.048
					Female	361.82 ± 40.86			
CCQ	Physical health	51–255	−0.241	0.93	Male	192.30 ± 31.55	1.355	0.176	0.127
					Female	188.57 ± 27.21			

Note. PBI = Parental Bonding Instrument, EQ-i = Bar-On Emotional Quotient Inventory, CCQ = Chinese Constitution Questionnaire, EI = Emotional intelligence.

**Table 3:** The correlation coefficients among the main variables.

	1	2	3	4	5	6	7
1 Parental overprotection	—						
2 Intrapersonal competence	−0.25 ***	—					
3 Interpersonal competence	−0.25 ***	0.67 ***	—				
4 Stress management	−0.25 ***	0.73 ***	0.49 ***	—			
5 Adaptability	−0.30 ***	0.82 ***	0.67 ***	0.78 ***	—		
6 Overall EI	−0.30 ***	0.94 ***	0.80 ***	0.84 ***	0.92 ***	—	
7 Physical health	−0.19 ***	0.47 ***	0.34 ***	0.45 ***	0.46 ***	0.49 ***	—

Note. EI = Emotional intelligence, *** *p* < .001 level (2-tailed).

**Table 4:** The hierarchical regression analysis of parental overprotection and physical health.

Variables	Physical Health									
	Model 1					Model 2				
	*B*	*SE*	*β*	*t*	*p*	*B*	*SE*	*β*	*t*	*p*
Age	1.627	0.961	0.079	1.693	0.091	2.259	0.952	0.110	2.371	0.018
Gender	−3.65	2.754	−0.062	−1.326	0.186	−3.198	2.700	−0.054	−1.184	0.237
Family SES	1.620	0.784	0.096	2.068	0.039	1.548	0.768	0.092	2.015	0.044
Parental overprotection						−0.522	0.117	−0.205	−4.449	<0.001
*F*	2.999					7.290				
Δ*F*	2.999					19.792				
*R* ^2^	0.019					0.060				
Δ*R*^2^	0.019					0.041				

**Table 5:** The hierarchical regression analysis of parental overprotection and EI.

Variables	EI									
	Model 1					Model 2				
	*B*	*SE*	*β*	*t*	*p*	*B*	*SE*	*β*	*t*	*p*
Age	0.51	1.398	0.017	0.365	0.716	1.865	1.349	0.062	1.382	0.168
Gender	1.415	4.006	0.016	0.353	0.724	2.385	3.826	0.028	0.623	0.533
Family SES	3.579	1.140	0.146	3.139	0.002	3.424	1.088	0.139	3.145	0.002
Parental overprotection						−1.122	0.166	−0.302	−6.744	<0.001
*F*	3.419					14.185				
Δ*F*	3.419					45.480				
*R* ^2^	0.022					0.111				
Δ*R*^2^	0.022					0.089				

Note. EI = Emotional intelligence, SES = socioeconomic status.

**Table 6:** The hierarchical regression analysis of EI and Physical health.

Variables	Physical Health									
	Model 1					Model 2				
	*B*	*SE*	*β*	*t*	*p*	*B*	*SE*	*β*	*t*	*p*
Age	1.627	0.961	0.079	1.693	0.091	1.392	0.842	0.068	1.654	0.099
Gender	−3.650	2.754	−0.062	−1.326	0.186	−3.075	2.475	−0.052	−1.242	0.215
Family SES	1.620	0.784	0.096	2.068	0.039	0.483	0.694	0.029	0.696	0.487
Intrapersonal competence						0.358	0.126	0.220	2.848	0.005
Interpersonal competence						0.107	0.165	0.038	0.647	0.518
Stress management						0.570	0.223	0.175	2.562	0.011
Adaptability						0.302	0.230	0.111	1.316	0.189
*F*	2.999					22.295				
Δ*F*	2.999					36.072				
*R* ^2^	0.019					0.257				
Δ*R*^2^	0.019					0.238				

Note. EI = Emotional intelligence, SES = socioeconomic status.

**Table 7:** Moderated Mediation Analysis Results Taking Gender as a Moderator.

Variables	Model 1 (Overall EI)					Model 2 (Physical Health)				
	*b*	SE	*t*	*p*	95% CI	*b*	SE	*t*	*p*	95% CI
Parental overprotection	−1.102	0.165	−6.675	<0.001	[−1.426, −0.777]	−0.129	0.110	−1.172	0.242	[−0.344, 0.869]
Overall EI						0.328	0.030	11.101	<0.001	[0.270, 0.386]
Gender	2.629	3.813	0.689	0.491	[−4.864, 10.122]	−4.343	2.403	−1.807	0.071	[−9.064, 0.379]
Parental overprotection × Gender	0.912	0.330	2.762	0.006	[0.263, 1.560]	0.015	0.219	0.070	0.944	[−0.415, 0.446]
Overall EI × Gender						0.013	0.059	0.215	0.830	[−0.103, 0.129]
*F*	17.411					29.918				
*R* ^2^	0.103					0.248				

Note. EI = Emotional intelligence.

## Data Availability

Data will be made available on https://osf.io/8upxj/overview?view_only=abe26bff05984ca38343b79b2b2081f8 (accessed on 10 December 2025).
